# Time Series Analysis of the Effectiveness and Safety of Capsule Endoscopy between the Premarketing and Postmarketing Settings: A Meta-Analysis

**DOI:** 10.1371/journal.pone.0153662

**Published:** 2016-06-01

**Authors:** Kazuo Iijima, Mitsuo Umezu, Kiyotaka Iwasaki

**Affiliations:** 1 Cooperative Major in Advanced Biomedical Sciences, Joint Graduate School of Tokyo Women's Medical University and Waseda University, Tokyo, Japan; 2 Faculty of Science and Engineering, Waseda University, Tokyo, Japan; University Hospital Llandough, UNITED KINGDOM

## Abstract

**Background:**

Clinical studies for assessing the effectiveness and safety in a premarketing setting are conducted under time and cost constraints. In recent years, postmarketing data analysis has been given more attention. However, to our knowledge, no studies have compared the effectiveness and the safety between the pre- and postmarketing settings. In this study, we aimed to investigate the importance of the postmarketing data analysis using clinical data.

**Methods and Findings:**

Studies on capsule endoscopy with rich clinical data in both pre- and postmarketing settings were selected for the analysis. For effectiveness, clinical studies published before October 10, 2015 comparing capsule endoscopy and conventional flexible endoscopy measuring the detection ratio of obscure gastrointestinal bleeding were selected (premarketing: 4 studies and postmarketing: 8 studies) from PubMed (MEDLINE), Cochrane Library, EMBASE and Web of Science. Among the 12 studies, 5 were blinded and 7 were non-blinded. A time series meta-analysis was conducted. Effectiveness (odds ratio) decreased in the postmarketing setting (premarketing: 5.19 [95% confidence interval: 3.07–8.76] vs. postmarketing: 1.48 [0.81–2.69]). The change in odds ratio was caused by the increase in the detection ratio with flexible endoscopy as the control group. The efficacy of capsule endoscopy did not change between pre- and postmarketing settings. Heterogeneity (*I*^2^) increased in the postmarketing setting because of one study. For safety, in terms of endoscope retention in the body, data from the approval summary and adverse event reports were analyzed. The incidence of retention decreased in the postmarketing setting (premarketing: 0.75% vs postmarketing: 0.095%). The introduction of the new patency capsule for checking the patency of the digestive tract might contribute to the decrease.

**Conclusions:**

Effectiveness and safety could change in the postmarketing setting. Therefore, time series meta-analyses could be useful to continuously monitor the effectiveness of medical device in clinical practices.

## Introduction

The global medical device market has a value of more than US$150 billion, with the US, Europe, and Japan having more than 65% of the market share [1]. Many medical devices are approved and marketed every year. Many clinical studies of medical devices are conducted in the pre- and postmarketing settings. The main objective of premarketing clinical studies is the approval for marketing. The study design and results are not necessarily matched with real world clinical outcomes because studies are conducted among limited doctors and patients because of time and cost constraints in premarketing settings. Thus, in the premarketing setting, evaluating the actual effectiveness and safety of medical devices is difficult [2, 3]. In contrast, after marketing, additional clinical studies are conducted with a larger number of physicians and patients. The analysis of postmarketing data enables evaluation of the effectiveness and safety of medical devices in real world clinical situations.

In recent years, postmarketing data has received more attention and several studies have been conducted [4–6]. Postmarketing surveillance complements the limited premarketing evaluation [4]. For constructing future fast approval systems, evaluation of the postmarketing data is considered to be one of the most important issues. Improvement in postmarketing systems will compensate for the lack of data on premarketing safety outcomes [5]. Systematic postmarketing data collection will provide the actual outcomes for clinicians [6]. However, these studies are limited with regards to collecting postmarketing data. To our knowledge, there are no studies to compare the effectiveness and safety of the pre- and postmarketing settings. Therefore, the aim of this study was to investigate the importance of the postmarketing data analysis using clinical data.

## Materials and Methods

### Selection of a research object

In this study, the capsule endoscope was selected because the amount of comparative study data in the pre- and postmarketing settings is rich for the capsule endoscope because it was initially approved as an unprecedented device in the Class II medical device classification and many clinical studies have been conducted. This device was developed for the detection of obscure gastrointestinal bleeding (OGIB). The dimension of this device is 27 × 11 mm and a CMOS camera and light-emitting diode (LED) illumination are located in front of the capsule. The capsule is propelled through the digestive tract via peristalsis, capturing approximately 60,000 digital images; it is excreted naturally in approximately 6 hours [7]. In the use of the flexible endoscope, patients are generally sedated in Europe and the US, but not sedated in Japan. The “bowel cleaning” process is required for both capsule and flexible endoscopy.

### Definition of the pre- and postmarketing periods

**Fig 1:**
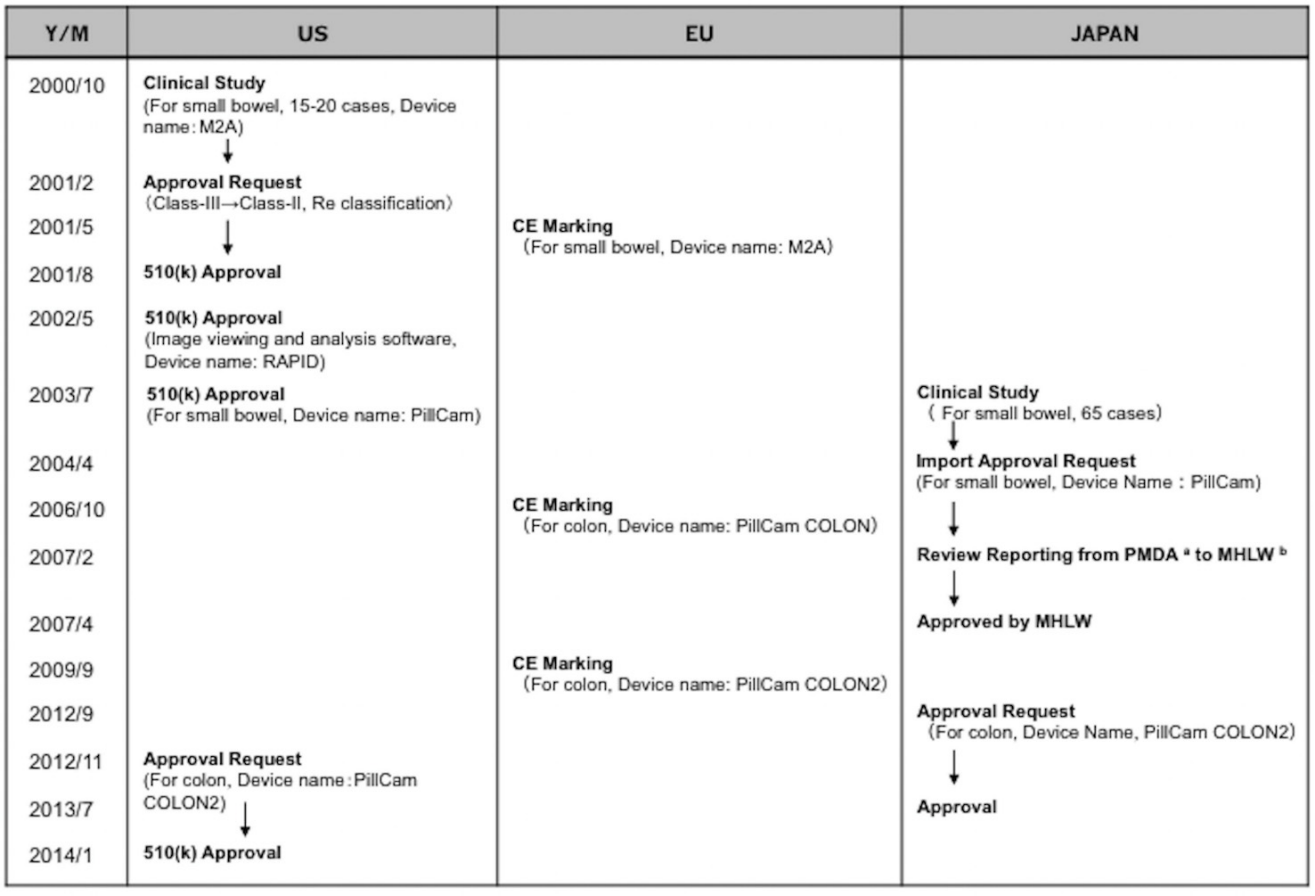
Approval history of capsule endoscope in the US, EU, and Japan. ^a^ Pharmaceutical and Medical Device Agency, Japan; ^b^ Health, Labour and Welfare Ministry.

The typical regional approval status of capsule endoscopy is shown in Fig 1. In this study, we defined the postmarketing setting as the period after 2003, the year that Food and Drug Administration approval was obtained.

### Effectiveness

#### Literature search and study selection

The evaluation outcome for effectiveness was the diagnostic yield of obscure gastrointestinal bleeding (OGIB) comparing the capsule endoscopy and conventional flexible endoscopy. Following the Preferred Reporting Items for Systematic Reviews and Meta-Analyses (PRISMA) guidelines [8] for meta-analyses, a literature search was performed for English articles published before October 10, 2015 using PubMed (MEDLINE), Cochrane Library, EMBASE and Web of Science. The initial search was performed by one reviewer (K. Iijima). The search keywords used were: “capsule endoscopy”, “capsule endoscope”, “PillCam”, and “M2M” in article titles (GIVEN Imaging Co. Ltd, premarketing: M2M, postmarketing: PillCam). To prevent missing potentially relevant studies, the initial search was not limited to OGIB. After the initial search, one reviewer (K. Iijima) screened all of the abstracts. The studies were divided into the premarketing and postmarketing groups based on the study period. The inclusion criterion was that the study compared capsule endoscopy and conventional flexible endoscopy in the same patients with OGIB. The exclusion criteria were studies without a focused diagnosis, OGIB detection, and non-comparative studies. Studies overlapping the pre- and postmarketing period were excluded. Studies comparing OGIB detection between capsule endoscopy and flexible endoscopy with different patients were excluded. Case studies, reviews, meta-analyses, and systematic reviews were also excluded. The studies selected were reviewed as full texts and independently assessed by two reviewers (K. Iijima and K. Iwasaki); any disagreement was resolved by consensus. We did not contact the authors of the original studies.

#### Quality assessment

In the present study, we evaluated the quality of the selected research studies using the Standards for Reporting of Diagnostic Accuracy (STARD) guidelines, which consisted of 25 items. Each paper was scored one point per item, and the total score was calculated. The quality of each study was assessed based on components of the Cochrane risk of bias tool [9]. All risk of bias assessments were conducted by two reviewers (K. Iijima and K. Iwasaki) and any disagreement was resolved by consensus.

#### Data extraction and analysis

The odds ratio of the diagnostic yield was examined as a measure of the outcome to compare the effectiveness between capsule endoscopy and conventional flexible endoscopy. In the selected study, the number of patients diagnosed with OGIB and the total number of patients who received capsule endoscopy or conventional flexible endoscopy were examined. Meta-analysis was performed with the Review Manager software (Review Manager version 5.2, The Cochrane Collaboration). The pooled data were weighted using the Mantel-Haenszel method. The random effects model was selected to calculate the estimated odds ratio and 95% confidence intervals (95%CI). The heterogeneity of the studies was assessed using the *I*^*2*^ test. Values of 0–25%, 25–50%, 50–75% and >75% were considered to represent low, moderate, high and very high diverseness, respectively [10]. The cumulative odds ratio and heterogeneity of the studies was calculated year-by-year. The data including and excluding the premarketing settings were analyzed. The subgroup analyses of the regional difference of studies were conducted by dividing studies into the two groups: (1) Europe (EU)/North America (US) and (2) Japan.

#### Analysis of heterogeneity

Each study from a total of 8 postmarketing studies was excluded from the meta-analysis one-by-one, and the corresponding heterogeneity was calculated.

### Safety

In order to evaluate changes in the safety of capsule endoscopy, we focused on the incidence of capsule endoscope retention in the body, which is reported to be the most frequently encountered adverse event in clinical cases. We compared data of the retention in the US, EU and Japan using several data sources (premarketing: Approval report of GIVEN Imaging capsule endoscope [11] and a published article [12]; postmarketing: Approval report of GIVEN Imaging capsule endoscope [11] and data of adverse events from the Ministry of Health, Labour and Welfare of Japan [13]). Both pre- and postmarketing data were available from the same data source in Japan. However, premarketing data in the US and EU and postmarketing data of EU were not available. The postmarketing data were investigated using the data of the postmarketing surveillance system (PMS) in the US and Japan.

## Results

### Effectiveness

#### Analysis of research papers

**Fig 2:**
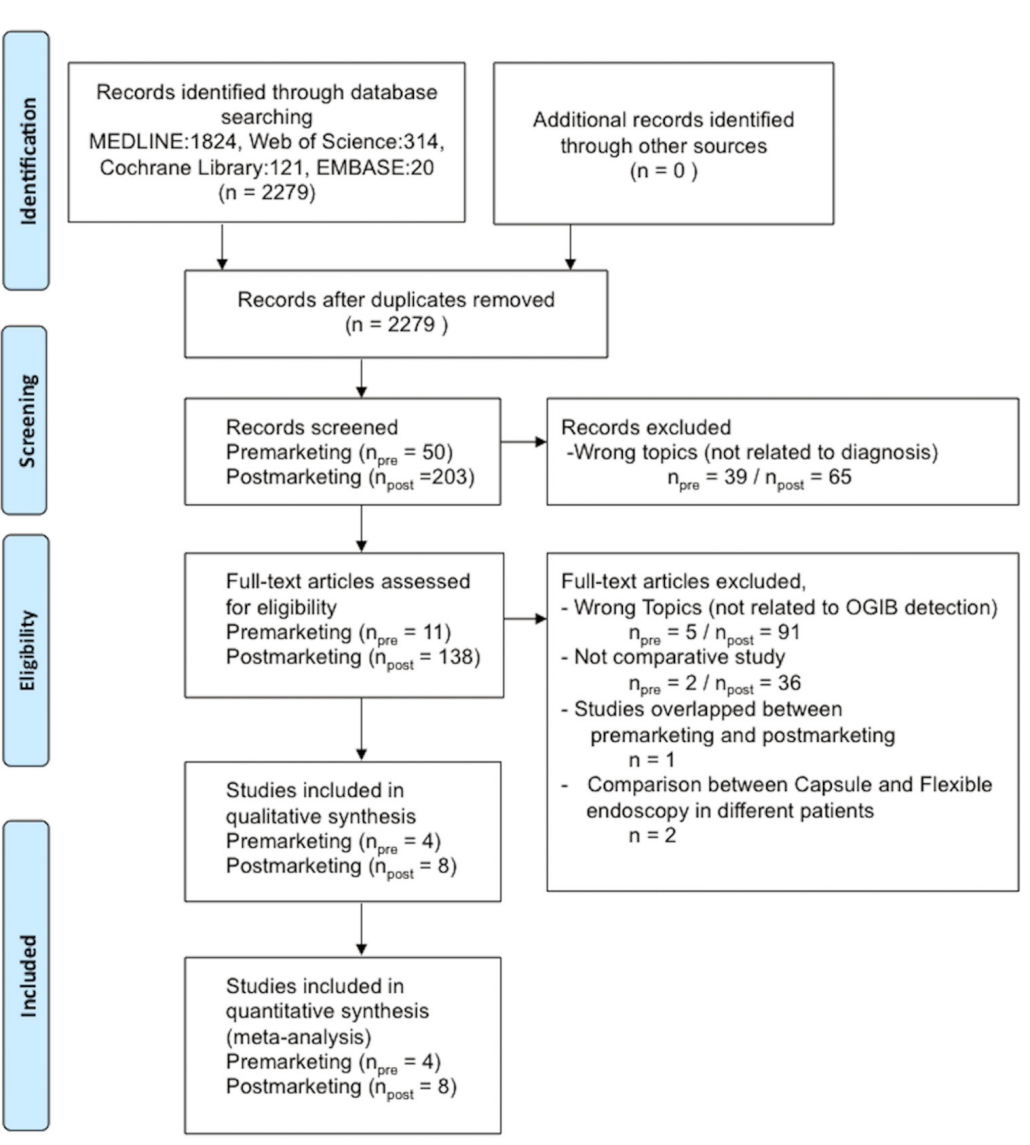
Flow chart of study selection for the meta-analysis.

The selection process of the included studies is shown in Fig 2. On the initial search, 50 premarketing and 203 postmarketing studies were extracted. Studies not related to diagnosis (premarketing, 39 studies, postmarketing, 65 studies) were excluded. Studies not related to OGIB detection (premarketing, 5 studies and postmarketing, 91 studies) were excluded. Non-comparative studies (premarketing, 2 studies and postmarketing, 36 studies) were excluded. One study that overlapped between the pre- and postmarketing period was excluded. Two studies that compared OGIB detection between capsule endoscopy and flexible endoscopy in different patients were excluded. Finally, 4 premarketing and 8 postmarketing studies were included in the meta-analysis [14–25].

The detailed data and quality assessment of the STARD score are shown in Table 1. Cochrane risk of bias summary is shown in Table 2. The mean STARD score was 16.0. The risk of bias was marked using the Index test and Reference standard. The countries in which the premarketing studies were performed were the US, Germany, and UK. The postmarketing studies were performed in the US, Italy, the Netherlands, and Japan. In particular, the number of postmarketing studies in Japan has increased over time. The number of patients tended to increase in the postmarketing setting.

**Table 1:** Data of the selected studies selected for the meta-analysis.

Phase	Study	Publish Year	Study Period	Country	Design	Statistical Analysis Method	Positive Capsule Endoscopy	Positive Flexible Endoscopy	STARD Score
**Pre**	Ell et al. [14]	2002	2001.4–2001.10	Germany	Prospective, Not blinded, Not randomized	Student's *t* test	21/32 (66%)	9/32 (28%)	15
	Lewis et al. [15]	2002	2000.10–2001.1	USA	Prospective, Not blinded, Not Randomized	McNemar test	11/20 (55%)	6/20 (30%)	12
	Hartmann et al. [16]	2003	2001.7–2002.11	Germany	Prospective, Blinded, Not Randomized	No statistical analysis	25/33 (76%)	7/33 (21%)	16
	Mylonaki et al. [17]	2003	1999–2000	UK	Prospective, Blinded, Not Randomized	χ^2^ test	34/50 (68%)	16/50 (32%)	17
**Post**	Matsumoto et al. [18]	2005	2004.4–2005.1	Japan	Prospective, Blinded, Not Randomized	No statistical analysis	10/13 (76.9%)	6/13 (46%)	13
	Mehdizadeh et al. [19]	2006	2004.8–2005.8	USA	Prospective, Not blinded, Not Randomized	Fisher's exact test	63/115 (54.8%)	57/115 (49.6%)	17
	Hadithi et al. [20]	2006	2003.11–2004.12	Netherland	Prospective, Not blinded, Not Randomized	Student's *t* test	28/35 (80%)	21/35 (60%)	20
	Kameda et al. [21]	2008	2005.4–2006.2	Japan	Prospective, Blinded, Not Randomized	χ^2^ test	23/32 (71.9%)	21/32 (65.6%)	16
	Marmo et al. [22]	2009	2004.1–2007.10	Italy	Prospective, Not blinded, Not Randomized	Kappa statistic	174/193 (90.2%)	132/193 (68.4%)	17
	Fukumoto et al. [23]	2009	2006.4–2007.9	Japan	Prospective, Blinded, Not Randomized	McNemar test	16/42 (38.1%)	18/42 (42.9%)	15
	Arakawa et al. [24]	2009	2004.6–2007.2	Japan	Retrospective, Not blinded, Not Randomized	McNemar test	40/74 (54.1%)	47/74 (63.5%)	15
	Shishido et al. [25]	2012	2006.4–2009.12	Japan	Prospective, Not blinded, Not Randomized	McNemar test	25/54 (46.3%)	28/54 (51.9%)	19

**Table 2:** Cochran risk of bias summary for the meta-analysis (QUADAS-2).

Study	RISK OF BIAS				APPLICABILITY CONCERNS		
	PATIENT SELECTION	INDEX TEST	REFERENCE STANDARD	FLOW AND TIMING	PATIENT SELECTION	INDEX TEST	REFERENCE STANDARD
Ell et al. [14]	L	U	L	L	L	L	L
Lewis et al. [15]	L	H	H	U	L	L	L
Hartmann et al. [16]	L	L	L	L	L	L	L
Mylonaki et al. [17]	L	L	L	L	L	L	L
Matsumoto et al. [18]	L	L	L	U	L	L	L
Mehdizadeh et al. [19]	L	L	U	U	L	L	L
Hadithi et al. [20]	L	H	H	L	L	L	L
Kameda et al. [21]	L	L	L	L	L	L	L
Marmo et al. [22]	L	L	H	L	L	L	L
Fukumoto et al. [23]	L	L	L	U	L	L	L
Arakawa et al. [24]	L	H	L	L	L	L	L
Shishido et al. [25]	L	L	H	L	L	L	L

L: Low Risk, H: High Risk, U: Unclear

#### Meta-analysis results

**Fig 3:**
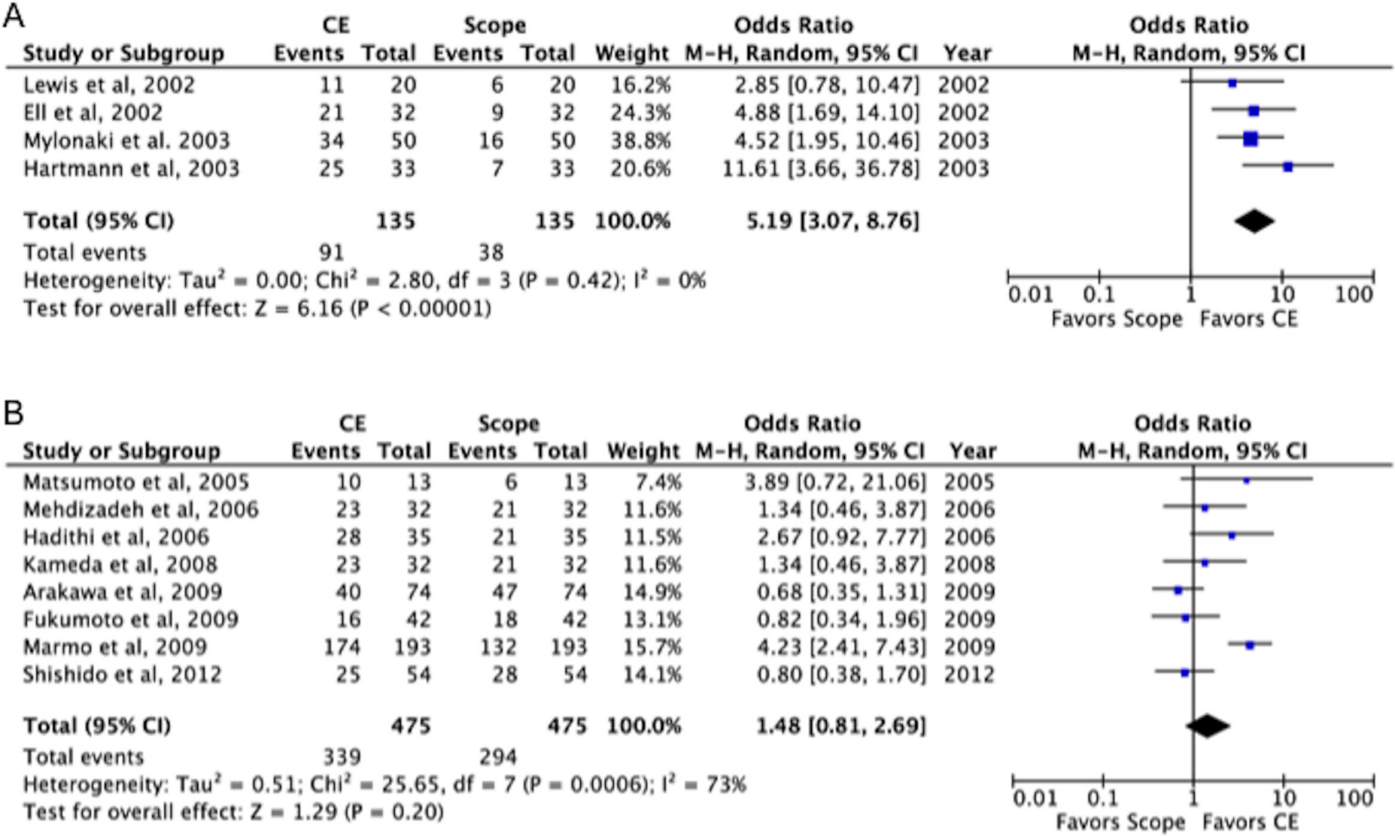
Forest plot of the odds ratio of OGIB detection in the premarketing and postmarketing settings. A: Premarketing setting. B: Postmarketing setting.

The results of the meta-analysis are shown in Fig 3. The odds ratios for effectiveness in the premarketing and postmarking settings were 5.19 (95% CI: 3.07–8.76) and 1.48 (95% CI: 0.81–2.69), respectively, indicating a decrease in the postmarketing studies. The heterogeneity (*I*^2^) values were 0% and 73%, respectively, indicating an increase in postmarketing studies.

**Fig 4:**
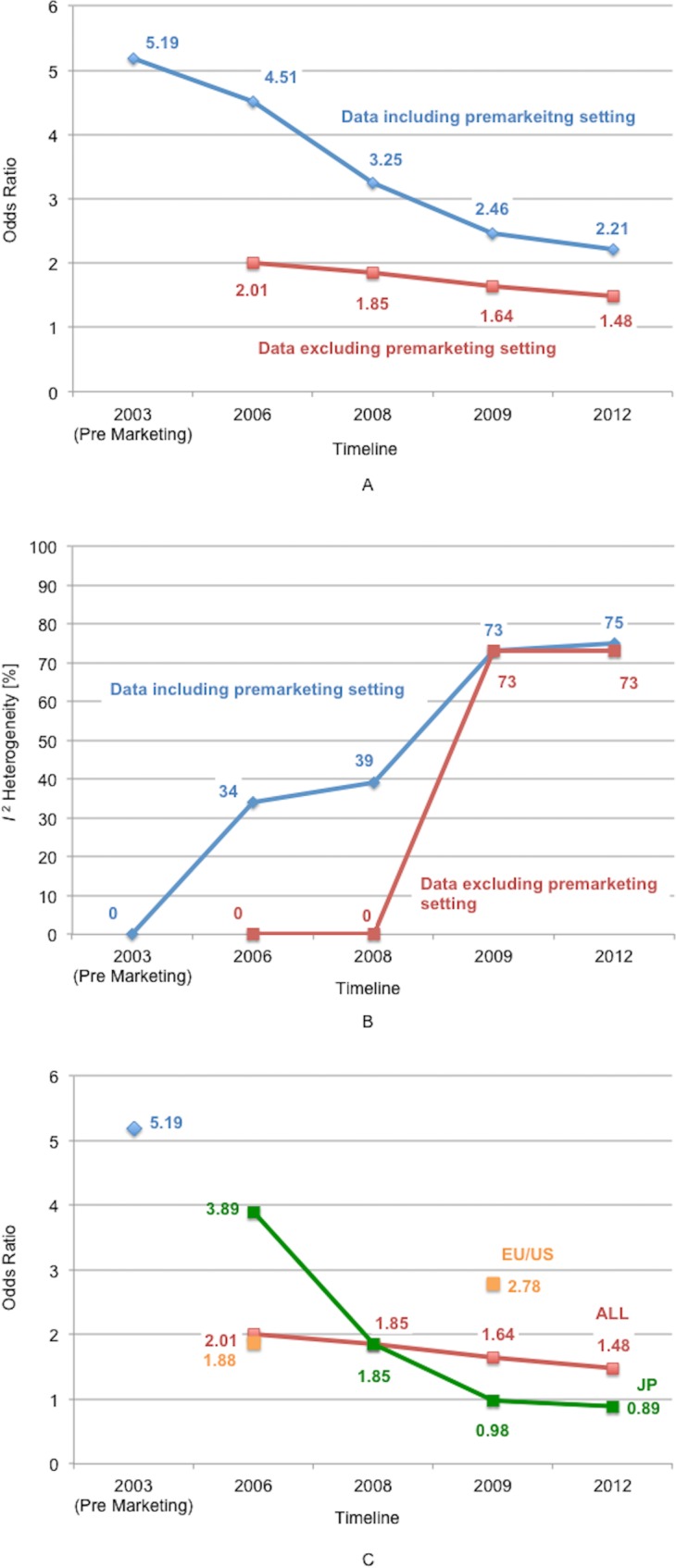
Changes in odds ratio and heterogeneity according to the time series. A: Odds ratio: Comparison between including and excluding premarketing data. B: Heterogeneity. C: Odds ratio: Subgroup analyses of the regional difference of studies.

The time series variations in odds ratio and heterogeneity are shown in Fig 4. The odds ratio showed a decreasing trend (Fig 4A) and heterogeneity showed an increasing trend with time (Fig 4B). Moreover, both the odds ratio and heterogeneity including the premarketing setting increased compared to the data excluding the premarketing setting. The postmarketing odds ratio in all countries decreased compared to the premarketing data. In the postmarketing setting, the odds ratio decreased in Japan and increased in the EU/US. In Japan in 2009, as the odds ratio decreased to less than one, flexible endoscopy became more effective than the capsule endoscopy.

#### Analysis of heterogeneity

**Fig 5:**
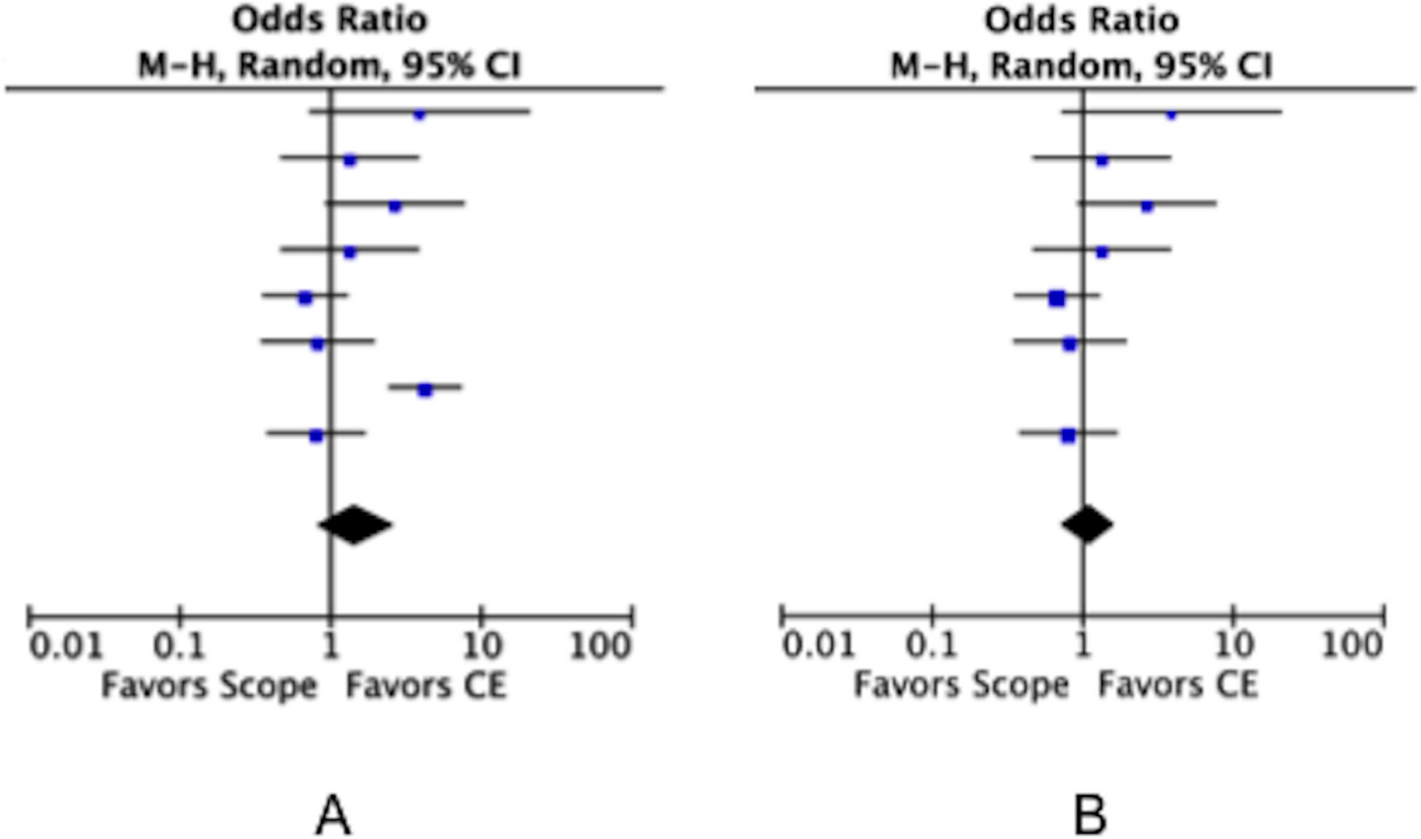
Forest plot of the odds ratio. A: Data including the overall studies. B: Data excluding the study of Marmo et al., [22].

The results of the heterogeneity analysis are shown in Table 3. The data from Marmo et al [22] in particular caused an increase in heterogeneity. When the data from Marmo et al were excluded from the analysis, the heterogeneity was distinctly decreased (heterogeneity including the data from Marmo et al: *I*^*2*^ = 73% vs heterogeneity excluding the data from Marmo et al: *I*^*2*^ = 26%). The odds ratio also decreased from 1.48 (95% CI: 0.81–2.69) to 1.01 (95% CI: 0.73–1.66; Fig 5).

**Table 3:** Changes in the heterogeneity when each postmarketing study is excluded.

Expected Data	Heterogeneity %	Increase or Decrease from Original
Matsumoto et al. [18]	75	2
Mehdizadeh et al. [19]	77	4
Hadithi et al. [20]	76	3
Kameda et al. [21]	77	4
Marmo et al. [22]	26	-47
Fukumoto et al. [23]	74	1
Arakawa et al. [24]	67	-6
Shishido et al. [25]	73	0

Overall heterogeneity of 8 studies: 73%

### Safety

Data of the retention of the capsule endoscope in the body were examined using the approval summary and published articles [11–13]. The premarketing values were 7.7% (Japan) [11] and 0.75% [12] (worldwide), and the postmarketing values were 4.6% (Japan) [13] and 0.095% (US) [13]. We found a large difference in the incidence of retention between Japan, worldwide, and the US. The incidence of retention in Japan tended to decrease in the postmarketing setting.

The results of the incidence of retention by using the database of the Ministry of Health, Labour and Welfare of Japan from October 2007 to September 2013 showed that the main adverse events were endoscope retention (70 cases), aspiration (11 cases), intestinal obstruction (6 cases), perforation (3 cases), and device failure (1 case).

## Discussion

### Effectiveness

#### Comparative effectiveness of capsule versus flexible endoscopy in the pre- and postmarketing settings

The effectiveness of capsule versus flexible endoscopy with respect to the OGIB detection was higher in the premarketing setting and decreased in the postmarketing setting. We examined percentages of OGIB detection of capsule and flexible endoscopy. The average diagnostic yield of the capsule endoscopy did not change between the pre- and postmarketing settings (premarketing, 66.3% (95% CI: 52.4%–80.0%); postmarketing, 64.0% (95% CI: 48.7%–79.3%), P = 0.83, t-test), while the diagnostic yield of the flexible endoscopy in the postmarketing setting increased (premarketing, 27.8% (95% CI: 20.1%–35.3%), postmarketing, 56.0% (95% CI: 47.9%–64.0%), P < 0.001, t-test). These data indicated that the efficacy of capsule endoscopy with respect to the detection of OGIB has been preserved between pre- and postmarketing settings, and the detection of OGIB of flexible endoscope has been improved in the postmarketing setting.

#### Potential factors affecting comparative effectiveness of capsule versus flexible endoscopy between the pre- and postmarketing settings

The odds ratio for the effectiveness of capsule endoscopy against flexible endoscopy significantly decreased in the postmarketing setting compared to the premarketing setting. The change may be explained by several factors beyond marketing approval. The type of flexible endoscope used differed between the pre- and postmarketing settings. In the premarketing studies, a push endoscope was available; whereas in the postmarketing studies, a double-balloon endoscope (Fujifilm Co. Ltd) was used because it was approved in 2004 in the US, and in 2003 in Japan. Japanese studies indicated that compared to the push endoscope, the double-balloon endoscope could be inserted deeper into the small intestine [26, 27]. Therefore, the detection ratio with flexible endoscopy might have increased due to the introduction of the double-balloon endoscope.

As shown in Table 1, 4 studies were conducted in EU and US in premarketing settings, and none in Japan. In contrast, 5 of 8 postmarketing studies were conducted in Japan. This might be because capsule endoscopy was antecedently developed in the EU/US and the flexible endoscope and double-balloon endoscope was developed in Japan and evaluated by many Japanese physicians. This regional difference might affect the odd ratios between the EU/US and Japan in the postmarketing setting (Fig 4C). The odds ratio of the Japanese group was lower than that of the EU/US group. Double-balloon endoscopy requires the skill of insertion, which is considered to be one of the strengths of Japanese endoscopy physicians. This may have affected the results of flexible endoscopy in the Japanese group in the postmarketing setting. The odds ratio associated with the EU/US was higher in 2009 than that in 2006. However, the tendency was not clear because only two-data-points were available.

#### Time series meta-analysis of postmarketing data

To our knowledge, no meta-analysis has been conducted according to the timeline of development. It is a standard approach to analyze all of the selected studies, regardless of the pre- or postmarketing nature. As shown in Fig 4A, even if the identical postmarketing data were used for the meta-analysis, the odds ratio and heterogeneity were estimated to be higher by including the premarketing data. This result implies the importance of excluding the premarketing data when the meta-analysis is performed with the aim of evaluating the real-world postmarketing situation. As shown in Fig 4C, the odds ratio of the Japanese study group changed with time.

#### Heterogeneity sensitivity analysis

**Fig 6:**
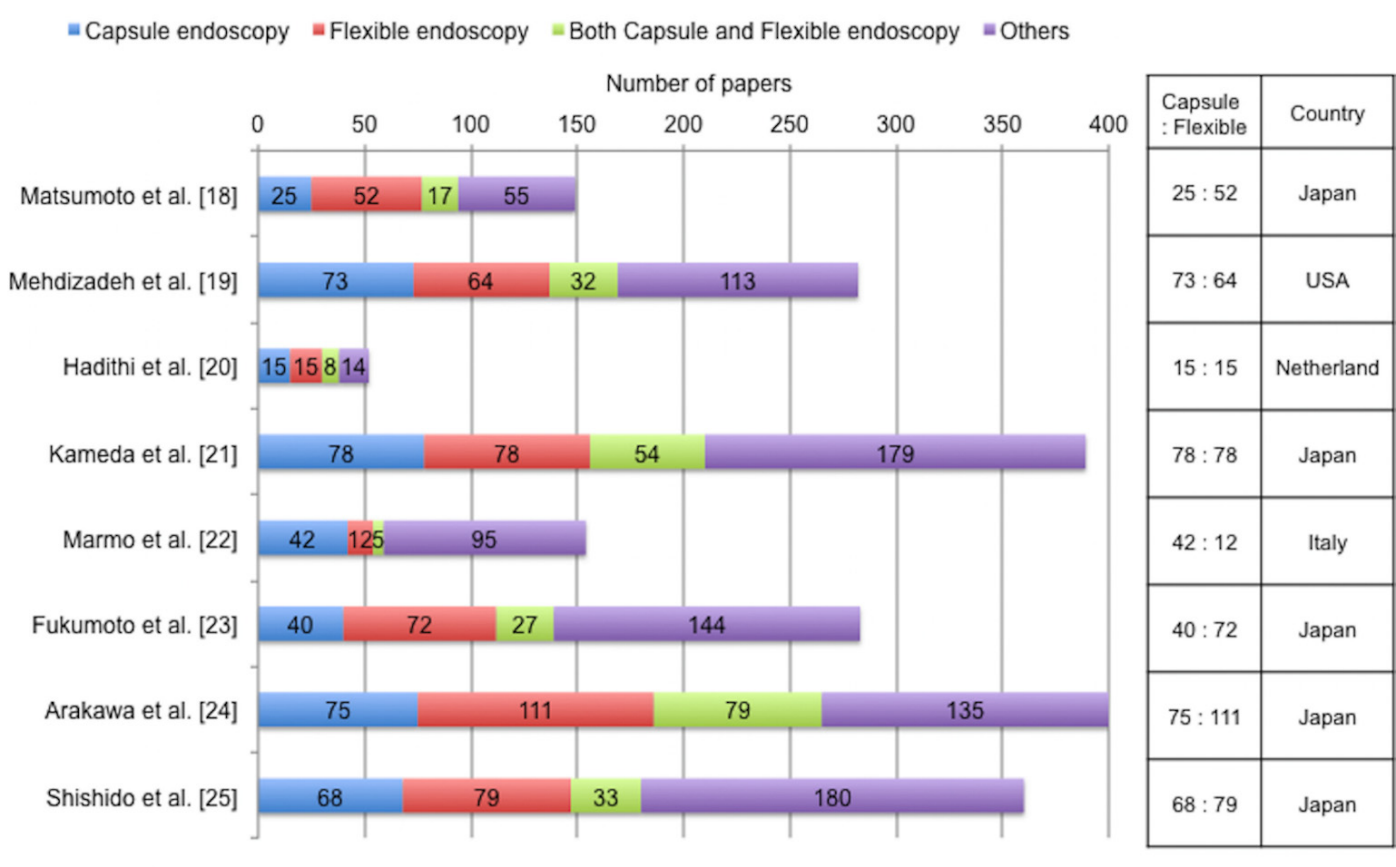
The number and categories of papers published by all authors of the eight postmarketing studies. A total of 2069 papers were found using PubMed (MEDLINE). The contents were summarized as the following four categories: capsule endoscopy, flexible endoscopy, both capsule and flexible endoscopy, and others not related to capsule or flexible endoscopy.

The heterogeneity sensitivity analysis showed that the data from Marmo et al affected the distinct increase in heterogeneity. That study was by far the largest in the postmarketing group. In an attempt to speculate potential reasons for the increased heterogeneity by Marmo et al, we examined papers published by all authors including co-authors of the 8 postmarketing studies using PubMed (MEDLINE). A total of 2069 papers were found. The contents were examined and summarized as the following 4 categories: capsule endoscopy, flexible endoscopy, both capsule and flexible endoscopy, and others not related to capsule or flexible endoscopy. As shown in Fig 6, the ratio of published papers regarding capsule to flexible endoscopy was the highest in the study by Marmo et al. It can be speculated that the Marmo group might be active in the research of capsule endoscopy. The anomalous result of the Marmo group might be explained by the pattern of papers published by this group.

### Safety

#### Changes in capsule endoscope retention ratio between pre- and postmarketing settings

The difference in the incidence of capsule endoscope retention among countries worldwide, the US, and Japan might be related to the study design. In the premarketing setting, the Japanese study was limited to patients that had stenosis due to Crohn’s disease. The difference in capsule endoscope retention between Japan (4.6%) and the US (0.095%) in the postmarketing setting might be caused by the timing of the introduction of the patency capsule [28]. The aim of the patency capsule is to check the condition of the stenosis prior to deciding whether to perform an endoscopy. This device was introduced earlier in the US (2006) than in Japan (2012), and this difference in the timing of approval might have caused the regional difference in retention of capsule endoscopy in the postmarketing setting between the US and Japan.

In terms of the change in retention ratio between the pre- and postmarketing settings, there could be several reasons for the differences in capsule endoscopic retention ratio between the US and Japan. In the US, capsule endoscopy was approved for use in patients with Crohn’s disease. However, in Japan, the device has been approved for use in patients without Crohn’s disease. The approval condition in Japan might have worked well for the decrease in the retention ratio from the pre- to the postmarketing setting. The reason for the decrease in the US postmarketing setting might be caused by the circumspect use of the capsule endoscope along with the combined use of the patency capsule.

#### Current status of safety data in the postmarketing setting

In the present study, we compare the safety of capsule endoscopy with respect to the incidence of endoscopic retention between the pre- and postmarketing settings in Japan because the data for both settings were available in the Approval [11] and Reevaluation reports [13]. The reevaluation system is unique to Japan and detailed postmarketing data are available in the reevaluation report. In addition, we obtained the postmarketing surveillance data of the US from these official documents in Japan, which are normally difficult to acquire.

Regarding the pre- and postmarketing data, in Japan, the Approval report and Reevaluation report are open-access official documents. In the US, the Approval report was open-access as the Approval Letter or Summary, but detailed data could not be obtained even under the Freedom of Information Act. We could obtain similar data limited to premarket approval application-approved devices and could not obtain data for the devices with 510(k) clearances. In the European Union, the detailed data are not open-access because of the self-certification system by the Notified Body, known as the CE marking.

Regarding the postmarketing data, there are no regional differences in the adverse event reporting system. However, in Japan, the Ministry of Health, Labour and Welfare constantly compiles incidence data and publishes a yearly summary report. In terms of information transparency, the Japanese system is active in disclosing information and provides favorable conditions for obtaining and examining postmarketing data.

### Limitations

This study has some limitations. First, 7 of the 12 studies were non-blinded where the same physician performed both capsule and flexible endoscopy. Second, publication bias might have affected the decreasing trend of the odds ratio. Third, this study was limited to capsule endoscopy.

## Conclusions

The findings of the present study indicated that efficacy of capsule endoscopy, in terms of OGIB detection, did not change between the pre- and the postmarketing settings. Meanwhile, the efficacy of flexible endoscopy increased possibly because of improvements in the postmarketing setting. Thus, the effectiveness of capsule endoscopy versus flexible endoscopy showed a decrease from the pre- to the postmarketing settings. The present study also indicated that, in terms of retention ratio, the safety of capsule endoscopy increased from that during the pre- to that during the postmarketing settings. This might have been a consequence of the approval condition and introduction of the patency capsule to pre-investigate the potential safety of capsule endoscopy before its use in each patient. This study implied that the effectiveness and safety of the medical device, in comparison with the control, may change between the pre- and postmarketing settings because of evolving test and/or control groups, possibly geographically varying physician experience, and the different approval times and approval conditions in different nations. Time series meta-analysis might be useful to continuously monitor the effectiveness of the medical device compared with the control. Further studies for other, various types of medical devices will contribute to our understanding of important factors that influence the benefit-risk balance of medical devices in real-world clinical practices.

## Supporting Information

S1 PRISMA Checklist (DOC)

S1 STARD Checklist (DOC)

S1 Table List of the full texts excluded. (DOCX)

## References

[1] Patel PB, Thula KC, Maheshwari DG. Medical device regulation and its comparison in Europe, Australia and India. Indo American Journal of Pharm Research. 2015;5(03):1211–22

[2] Food and Drug Administration. Balancing premarket and postmarket data collection for devices subject to premarket approval. CDRH Report. 2015 4

[3] Kramer DB, Mallis E, Zuckerman BD, Zimmerman BA, et al Premarket clinical evaluation of novel cardiovascular device. Am J Ther. 2012;17:2–7 10.1097/MJT.0b013e3181ca8105 20038828

[4] Resnic FS, Normand SL. Postmarketing Surveillance of Medical Device–Filling in the Gaps. Engl J Med. 2012;366(10):875–7 10.1056/NEJMp1114865 22332950

[5] Kramer DB, Xu S, Kesselheim AS. How Dose Medical Device Regulation Perform in the United States and the European Union? A Systematic Review. PLoS Medicine 2012;9(7):1–9 10.1371/journal.pmed.1001276 PMC3418047 22912563

[6] Sorenson C., Drummond M. Improving Medical Device Regulation: The United States and Europe in Perspective. The Milbank Quarterly. 2014;92(1):114–150 10.1111/1468-0009.12043 24597558 PMC3955380

[7] Eliakim AR. Video capsule endoscopy of the small bowel (PillCam SB). Current Opinion Gastroenterology. 2006;22(2):124–7 10.1097/01.mog.0000203866.25384.be 16462167

[8] Larissa S, David M, Mike C, Davina G, Alessandro L, Mark P, et al Preferred reporting items for systematic review and meta-analysis protocols (PRISMA-P) 2015: elaboration and explanation. BMJ 2015;349:g7647 10.1136/bmj.g7647 25555855

[9] Cochrane Handbook for Systematic Reviews of Interventions. 1st Edition. Julian P. T. Higgins, Sally Green, editor. Wiley(NJ);2008

[10] Higgins JPT, Altman DG, Gøtzsche PC, Jüni P, Moher D, et al The Cochrane Collaboration’s tool for assessing risk of bias in randomised trials. BMJ. 2011;343:d5928 10.1136/bmj.d5928 22008217 PMC3196245

[11] Pharmaceutical and Medical Device Agency, Japan. Approval report of capsule endoscope (GIVEN Imaging co. ltd), 7, 2007. Available: http://www.info.pmda.go.jp/nmdevices/M200700001/230424001_21900BZY00045000_A100_1.pdf

[12] Barkin JS, Friedman S. Wireless capsule endoscopy requiring surgical intervention: the world’s experience. Am J Gastroentrol. 2002;97:A83.

[13] Pharmaceutical and Medical Device Agency, Japan. Reevaluation report of capsule endoscope (GIVEN Imaging Co.), 2–3, 2013. Available: http://www.info.pmda.go.jp/saishinsa_kiki/M201300013/230424000_21900BZY00045000_100_1.pdf

[14] Ell C, Remke S, May A, Helou L, Henrich R, Mayer G. The first prospective controlled trial comparing wireless capsule endoscopy with push enteroscopy in chronic gastrointestinal bleeding. Endoscopy. 2002;34(9):685–9. 12195324 10.1055/s-2002-33446

[15] Lewis BS, Swain P. Capsule endoscopy in the evaluation of patients with suspected small intestinal bleeding: results of a pilot study. Gastrointest Endosc. 2002;56(3):349–53. 12196771 10.1016/s0016-5107(02)70037-0

[16] Hartmann D, Schilling D, Bolz G, Hahne M, Jakobs R, Siegel E, et al Capsule endoscopy versus push enteroscopy in patients with occult gastrointestinal bleeding. Z Gastroenterol. 2003;41(5):377–82. 12772049 10.1055/s-2003-39330

[17] Mylonaki M, Fritscher-Ravens A, Swain P. Wireless Wireless capsule endoscopy: a comparison with push enteroscopy in patients with gastroscopy and colonoscopy negative gastrointestinal bleeding. Gut 2003;52:1122–6 12865269 10.1136/gut.52.8.1122 PMC1773749

[18] Matsumoto T, Esaki M, Moriyama T, Nakamura S, Iida M. Comparison of capsule endoscopy and enteroscopy with the double-balloon method in patients with obscure bleeding and polyposis. Endoscopy. 2005;37(9):827–32. 16116533 10.1055/s-2005-870207

[19] Mehdizadeh S, Ross A, Gerson L, Leighton J, Chen A, Schembre D, et al What is the learning curve associated with double-balloon enteroscopy? Technical details and early experience in 6 US tertiary care centers. Gastrointest Endosc. 2006;64(5):740–50. 17055868 10.1016/j.gie.2006.05.022

[20] Hadithi M, Heine GD, Jacobs MA, van Bodegraven AA, Mulder CJ. A prospective study comparing video capsule endoscopy with double-balloon enteroscopy in patients with obscure gastrointestinal bleeding. Am J Gastroenterol. 2006;101:52–7. 16405533 10.1111/j.1572-0241.2005.00346.x

[21] Kameda N, Higuchi K, Shiba M, Machida H, Okazaki H, Yamagami H, et al A prospective, single-blind trial comparing wireless capsule endoscopy and double-balloon enteroscopy in patients with obscure gastrointestinal bleeding. J Gastroenterol. 2008;43(6):434–40. 10.1007/s00535-008-2182-9 18600387

[22] Marmo R, Rotondano G, Casetti T, Manes G, Chilovi F, Sprujevnik T, et al Degree of concordance between double-balloon enteroscopy and capsule endoscopy in obscure gastrointestinal bleeding: a multicenter study. Endoscopy. 2009;41(7):587–92. 10.1055/s-0029-1214896 19588285

[23] Fukumoto, Tanaka S, Shishido T, Takemura Y, Oka S, Chayama K. Comparison of detectability of small-bowel lesions between capsule endoscopy and double-balloon endoscopy for patients with suspected small-bowel disease. Gastrointest Endosc. 2009;69(4):857–64. 10.1016/j.gie.2008.06.007 19136103

[24] Arakawa D, Ohmiya N, Nakamura M, Honda W, Shirai O, Itoh A, et al Outcome after enteroscopy for patients with obscure GI bleeding: diagnostic comparison between double-balloon endoscopy and videocapsule endoscopy. Gastrointest Endosc. 2009;69(4):866–74. 10.1016/j.gie.2008.06.008 19136098

[25] Shishido T, Oka S, Tanaka S, Aoyama T, Watari I, Imagawa H, et al Diagnostic yield of capsule endoscopy vs. double-balloon endoscopy for patients who have undergone total enteroscopy with obscure gastrointestinal bleeding. Hepatogastroenterology. 2012;59(116):955–9. 10.5754/hge12242 22580642

[26] Yamamoto H, Sugano K. A new method of enteroscopy—the double-balloon method. Can J Gastroenterol. 2003;17(4):273–4. 12704472 10.1155/2003/309532

[27] Matsumoto T, Moriyama T, Esaki M, Nakamura S, Iida M. Performance of antegrade double-balloon enteroscopy: comparison with push enteroscopy. Gastrointest Endosc. 2005;62(3):392–8. 16111958 10.1016/j.gie.2005.04.052

[28] Spada C. A novel diagnostic tool for detecting functional patency of the small bowel: the Given patency capsule. Endoscopy. 2005;37(9):793–800. 16116528 10.1055/s-2005-870246

