# Supplementary material for: Time Series Analysis of the Effectiveness and Safety of Capsule Endoscopy between the Premarketing and Postmarketing Settings: A Meta-Analysis
Source: PLoS One. 2016 Jun 1;11(6):e0153662. doi: 10.1371/journal.pone.0153662 (PMC4889146; doi:10.1371/journal.pone.0153662)
Supplement: S1 Table — (DOCX) [file pone.0153662.s003.docx]

**S3. List of the full text excluded studies**

**Premarketing**

**1) Wrong Topics (no OGIB detected)**

|  | Author (Top) | Title | Journal | Reason of Exclusion |
| --- | --- | --- | --- | --- |
| 1 | Chong AK | Initial experience with capsule endoscopy at a major referral hospital. | Med J Aust. 2003 Jun 2;178(11):537-540 | The main outcome does not match. (Main outcome: all abnormal findings) |
| 2 | Liangpunsakul S | Wireless capsule endoscopy detects small bowel ulcers in patients with normal results from state of the art enteroclysis. | Am J Gastroenterol. 2003 Jun;98(6): 1295-1298 | The main outcome does not match. (Main outcome: ulcers findings) |
| 3 | Fireman Z | Diagnosing small bowel Crohn’s disease with wireless capsule endoscopy | Gut. 2003 Mar | Patients included in the study are limited. (Crohn’s disease) |
| 4 | Hartmann D | Diagnosis of a high-grade B-cell lymphoma of the small bowel by means of wireless capsule endoscopy | Z Gastroenterol. 2003 Feb;41(2):171-174 | The main outcome does not match. (Main outcome: B-cell lymphoma of the small bowel) |
| 5 | Costamagna G | A prospective trial comparing small bowel radiographs and video capsule endoscopy for suspected small bowel disease | Gastroenterology, 2002 Oct;123(4):999-1005 | Comparison with the barium follow-through method. |

**2) Not Comparative**

|  | Author (Top) | Title | Journal | Reason of Exclusion |
| --- | --- | --- | --- | --- |
| 1 | Scapa E | Initial experience of wireless-capsule endoscopy for evaluating occult gastrointestinal bleeding and suspected small bowel pathology. | Am J Gastroenterol. 2002 Nov;97(11):2776-2779 | Single arm study of the capsule endoscopy |
| 2 | Lewis BS | The advent of capsule endoscopy – a not-so-futuristic approach to obscure gastrointestinal bleeding. | Aliment Pharmacol Ther. 2003 May 1;17(9):1085-1096 | Review article |

**Postmarketing**

**1) Wrong Topics (no OGIB detected)**

|  | Author (Top) | Title | Journal | Reason of Exclusion |
| --- | --- | --- | --- | --- |
| 1 | Kobayashi Y | Efficacy of flexible spectral imaging color enhancement on the detection of small intestinal diseases by capsule endoscopy. | J Dig Dis. 2012 Dec;13(12):614-620 | The main outcome does not match. (Main outcome: Capsule endoscopy with new diagnostic technology-FICE) |
| 2 | Leung WK | Capsule endoscopy or angiography in patients with acute overt obscure gastrointestinal bleeding: a prospective randomized study with long-term follow-up. | Am J Gastroenterol. 2012 Sep;107(9):1370-6 | The main outcome does not match. (Main outcome: Patient burden) |
| 3 | Duque G | Virtual chromoendoscopy can be a useful software tool in capsule endoscopy. | Rev Esp Enferm Dig. 2012 May;104(5):231-236. | The main outcome does not match. (Main outcome: Capsule endoscopy with new diagnostic technology-FICE) |
| 4 | Agrawal JR | Diagnostic yield of dual-phase computed tomography enterography in patients with obscure gastrointestinal bleeding and a non-diagnostic capsule endoscopy. | J Gastroenterol Hepatol. 2012 Apr;27(4):751-759 | Comparing to CT scanning. |
| 5 | Long MD | Impact of capsule endoscopy on management of inflammatory bowel disease: a single tertiary care center experience. | Inflamm Bowel Dis. 2011 Sep;17(9):1855-1862 | Topic of a colon capsule endoscopy |
| 6 | Pioche M | Prospective, randomized comparison of two small-bowel capsule endoscopy systems in patients with obscure GI bleeding. | Gastrointest Endosc. 2011 Jun;73(6):1181-1188 | Comparing between two kinds of capsule endoscopes (PillCam vs MicroCam) |
| 7 | Spada C | Second-generation colon capsule endoscopy compared with colonoscopy. | Gastrointest Endosc. 2011 Sep;74(3):581-589.e1 | Topic of a colon capsule endoscopy |
| 8 | Hosono K | Optimal approach for small bowel capsule endoscopy using polyethylene glycol and metoclopramide with the assistance of a real-time viewer. | Digestion. 2011;84(2):119-125 | The main outcome does not match. (Main outcome: Imaging quality of Capsule endoscopy) |
| 9 | Petruzziello C | Small bowel capsule endoscopy vs conventional techniques in patients with symptoms highly compatible with Crohn's disease. | J Crohns Colitis. 2011 Apr;5(2):139-147 | Comparing to Ultrasonography. |
| 10 | Jensen MD | Diagnostic accuracy of capsule endoscopy for small bowel Crohn's disease is superior to that of MR enterography or CT enterography. | Clin Gastroenterol Hepatol. 2011 Feb;9(2):124-9 | Comparing to CT/MRI. |
| 11 | Sacher-Huvelin S | Colon capsule endoscopy vs. colonoscopy in patients at average or increased risk of colorectal cancer. | Aliment Pharmacol Ther. 2010 Nov;32(9):1145-53 | Topic of a colon capsule endoscopy |
| 12 | Liao Z | Completion rate and diagnostic yield of small-bowel capsule endoscopy: 1 vs. 2 frames per second. | Endoscopy. 2010 May;42(5):360-4 | The main outcome does not match. (Main outcome: Imaging Frame rate of Capsule endoscopy) |
| 13 | Laine L | Does capsule endoscopy improve outcomes in obscure gastrointestinal bleeding? Randomized trial versus dedicated small bowel radiography. | Gastroenterology. 2010 May;138(5):1673-1680.e1; quiz e11-2 | Comparing to Radiography |
| 14 | Hooks SB 3rd | Lubiprostone neither decreases gastric and small-bowel transit time nor improves visualization of small bowel for capsule endoscopy: a double-blind, placebo-controlled study. | Gastrointest Endosc. 2009 Nov;70(5):942-6 | The main outcome does not match. (Main outcome: Gastric transit time, small-bowel transit time and adequacy of small-bowel cleansing preparation) |
| 15 | Oette M | Wireless capsule endoscopy for the detection of small bowel diseases in HIV-1-infected patients. | Eur J Med Res. 2009 May 14;14(5):191-4 | Patients included in the study are limited. (HIV-1-infected patients) |
| 16 | Endo H | Characteristics of small bowel injury in symptomatic chronic low-dose aspirin users: the experience of two medical centers in capsule endoscopy. | J Gastroenterol. 2009;44(6):544-9 | Patients included in the study are limited. (Low-Dose-Aspirin applied patients) |
| 17 | Ohlsson B | A prospective evaluation of the diagnostic value of video capsule endoscopy in patients initially classified as irritable bowel syndrome. | Eur J Intern Med. 2009 Jan;20(1):48-52 | Patients included in the study are limited. (Irritable bowel syndrome) |
| 18 | Albert JG | Impact of capsule endoscopy on outcome in mid-intestinal bleeding: a multicentre cohort study in 285 patients. | Eur J Gastroenterol Hepatol. 2008 Oct;20(10):971-7 | Comparing to Radiography |
| 19 | Frenette CT | Comparison of esophageal capsule endoscopy and esophagogastroduodenoscopy for diagnosis of esophageal varices. | World J Gastroenterol. 2008 Jul 28;14(28):4480-4485 | Comparing to esophagogastroduodenoscopy only. |
| 20 | Goulas S | Capsule endoscopy in the investigation of patients with portal hypertension and anemia. | Can J Gastroenterol. 2008 May;22(5):469-474 | Patients included in the study are limited. (Patients with portal hypertension) |
| 21 | de Franchis R | Esophageal capsule endoscopy for screening and surveillance of esophageal varices in patients with portal hypertension. | Hepatology. 2008 May;47(5):1595-1603 | Patients included in the study are limited. (Patients with esophageal) |
| 22 | Hartmann D | Diagnosis of small-bowel pathology using paired capsule endoscopy with two different devices: a randomized study. | Endoscopy. 2007 Dec;39(12):1041-5 | Comparing between two kinds of capsule endoscopes (Given PillCam SB and Olympus EndoCapsule) |
| 23 | Delvaux M | Esophageal capsule endoscopy in patients with suspected esophageal disease: double blinded comparison with esophagogastroduodenoscopy and assessment of interobserver variability. | Endoscopy. 2008 Jan;40(1):16-22 | Comparing to Radiography esophagogastroduodenoscopy only. |
| 24 | Hoog CM | Findings in patients with chronic intestinal dysmotility investigated by capsule endoscopy. | BMC Gastroenterol. 2007 Jul 18;7:29. | Patients included in the study are limited. (Patients with neuropathic and one with indeterminate Chronic Intestinal Dysmotility.) |
| 25 | May A | Prospective multicenter trial of capsule endoscopy in patients with chronic abdominal pain, diarrhea and other signs and symptoms (CEDAP-Plus Study). | Endoscopy. 2007 Jul;39(7):606-12. | Patients included in the study are limited. (Patients with e chronic abdominal pain, plus other symptoms singly or in combination) |
| 26 | Sharma P | The diagnostic accuracy of esophageal capsule endoscopy in patients with gastroesophageal reflux disease and Barrett's esophagus: a blinded, prospective study. | Am J Gastroenterol. 2008 Mar;103(3):525-532 | The main outcome does not match. (Main outcome: Barrett's esophagus (BE), erosive esophagitis, and hiatal hernia) |
| 27 | Park CH | Utility of capsule endoscopy for the classification of Crohn's disease: a multicenter study in Korea. | Dig Dis Sci. 2007 Jun;52(6):1405-9 | Patients included in the study are limited. (Patients with Crohn’s disease) |
| 28 | Shiotani A | Visualization of the small intestine using capsule endoscopy in healthy subjects. | Dig Dis Sci. 2007 Apr;52(4):1019-25 | The main outcome does not match. (Main outcome: Preparation method of capsule endoscopy) |
| 29 | Neumann S | Wireless capsule endoscopy for diagnosis of acute intestinal graft-versus-host disease. | Gastrointest Endosc. 2007 Mar;65(3):403-9. | Comparing to Radiography esophagogastroduodenoscopy only. |
| 30 | Maunoury V | Value of wireless capsule endoscopy in patients with indeterminate colitis (inflammatory bowel disease type unclassified). | Inflamm Bowel Dis. 2007 Feb;13(2):152-5. | Patients included in the study are limited. (Patients with inflammatory bowel disease type unclassified (IBDU)) |
| 31 | Girelli CM | Clinical outcome of patients examined by capsule endoscopy for suspected small bowel Crohn's disease. | Dig Liver Dis. 2007 Feb;39(2):148-54 | Patients included in the study are limited. (Patients with Crohn’s disease) |
| 32 | Shim KN | Abdominal pain accompanied by weight loss may increase the diagnostic yield of capsule endoscopy: a Korean multicenter study. | Scand J Gastroenterol. 2006 Aug;41(8):983-8. | The main outcome does not match. (Main outcome: Relationship between findings and weight loss) |
| 33 | De Bona M | Capsule endoscopy findings in patients with suspected Crohn's disease and biochemical markers of inflammation. | Dig Liver Dis. 2006 May;38(5):331-5 | Patients included in the study are limited. (Patients with Crohn’s disease) |
| 34 | Eisen GM | The accuracy of PillCam ESO capsule endoscopy versus conventional upper endoscopy for the diagnosis of esophageal varices: a prospective three-center pilot study. | Endoscopy. 2006 Jan;38(1):31-5. | Comparing to Radiography esophagogastroduodenoscopy only. |
| 35 | Marmo R | Capsule endoscopy versus enteroclysis in the detection of small-bowel involvement in Crohn's disease: a prospective trial. | Clin Gastroenterol Hepatol. 2005 Aug;3(8):772-6. | Patients included in the study are limited. (Patients with Crohn’s disease) |
| 36 | De Palma GD | Mucosal abnormalities of the small bowel in patients with cirrhosis and portal hypertension: a capsule endoscopy study. | Gastrointest Endosc. 2005 Oct;62(4):529-534. | Patients included in the study are limited. (Patients with cirrhosis and portal hypertension) |
| 37 | Neu B | Capsule endoscopy versus standard tests in influencing management of obscure digestive bleeding: results from a German multicenter trial. | Am J Gastroenterol. 2005 Aug;100(8):1736-42. | Comparing to Angiography. |
| 38 | Eliakim R | A prospective study of the diagnostic accuracy of PillCam ESO esophageal capsule endoscopy versus conventional upper endoscopy in patients with chronic gastroesophageal reflux diseases. | J Clin Gastroenterol. 2005 Aug;39(7):572-8. | Comparing to esophagogastroduodenoscopy only. |
| 39 | Mata A | A prospective trial comparing wireless capsule endoscopy and barium contrast series for small-bowel surveillance in hereditary GI polyposis syndromes. | Gastrointest Endosc. 2005 May;61(6):721-5. | Comparing to the barium follow-through method. |
| 40 | Chong AK | Capsule endoscopy vs. push enteroscopy and enteroclysis in suspected small-bowel Crohn's disease. | Gastrointest Endosc. 2005 Feb;61(2):255-261 | Patients included in the study are limited. (Patients with Crohn’s disease) |
| 41 | Schulmann K | Feasibility and diagnostic utility of video capsule endoscopy for the detection of small bowel polyps in patients with hereditary polyposis syndromes. | Am J Gastroenterol. 2005 Jan;100(1):27-37. | Patients included in the study are limited. (Patients with hereditary polyposis syndromes.) |
| 42 | Soares J | Wireless capsule endoscopy for evaluation of phenotypic expression of small-bowel polyps in patients with Peutz-Jeghers syndrome and in symptomatic first-degree relatives. | Endoscopy. 2004 Dec;36(12):1060-6. | Patients included in the study are limited. (Patients with Peutz-Jeghers syndrome) |
| 43 | Caspari R | Comparison of capsule endoscopy and magnetic resonance imaging for the detection of polyps of the small intestine in patients with familial adenomatous polyposis or with Peutz-Jeghers' syndrome. | Endoscopy. 2004 Dec;36(12):1054-9. | Comparing to MRI |
| 44 | Fireman Z | Capsule endoscopy in real life: a four-centre experience of 160 consecutive patients in Israel. | Eur J Gastroenterol Hepatol. 2004 Sep;16(9):927-931. | The main outcome does not match. (Main outcome: Determination of the indication for capsule endoscopy) |
| 45 | Gralnek IM | Capsule endoscopy in acute upper gastrointestinal hemorrhage: a prospective cohort study. | Endoscopy. 2013;45(1):12-9 | Comparing to esophagogastroduodenoscopy only. |
| 46 | Di Biase L | Esophageal capsule endoscopy after radiofrequency catheter ablation for atrial fibrillation: documented higher risk of luminal esophageal damage with general anesthesia as compared with conscious sedation. | Circ Arrhythm Electrophysiol. 2009 Apr;2(2):108-12 | The main outcome does not match. (Main outcome: Determination of the indication for capsule endoscopy) |
| 47 | Lapalus MG | Esophageal capsule endoscopy vs. EGD for the evaluation of portal hypertension: a French prospective multicenter comparative study. | Am J Gastroenterol. 2009 May;104(5):1112-8 | Comparing to esophagogastroduodenoscopy only. |
| 48 | Segarajasingam DS | Randomized controlled trial comparing outcomes of video capsule endoscopy with push enteroscopy in obscure gastrointestinal bleeding. | Canadian J Gastroenterology and Hepatology, 2015;29(2):85-90 | Difference of comparing device |
| 49 | Rayner-Hartley E | Comparison of moviprep, pico-salax and overnight fast in small bowel capsule endoscopy preparation. | Gastrointestinal endoscopy, 2014;79(5 SUPPL.1):AB210 | Comparing to Colonoscope |
| 50 | Xu H | Preparation method of capsule endoscopy before operation | Journal of gastroenterology and hepatology, 2013;28:479 | The main outcome does not match. (Main outcome: Evaluation of Preparation) |
| 51 | Leung WK | Capsule endoscopy or angiography in patients with acute overt obscure gastrointestinal bleeding: a prospective randomized study with long-term follow-up. | Am J Gastroenterol., 2012; 107(9):1370-6 | Comparing to Angiography |
| 52 | Leung WK | Immediate capsule endoscopy or angiogram in patients with acute obscure gastrointestinal bleeding: A prospective randomized study with long term follow up. | Gastrointestinal endoscop, 2012; 75(4)SUPPL.1: AB124-AB125 | Comparing to Angiography |
| 53 | Pioche M | Prospective, randomized comparison of two small-bowel capsule endoscopy systems in patients with obscure GI bleeding. | Gastrointestinal endoscopy, 2011;73(6): 1181-8 | Comparing between two kinds of capsule endoscopes (Given PillCam SB and Medivators MiroCam) |
| 54 | Rubin M | Live view video capsule endoscopy enables risk stratification of patients with acute upper GI bleeding in the emergency room: a pilot study. | Digestive diseases and sciences, 2011; 56(3):786-791 | Emergency case only |
| 55 | Huprich JE | Prospective blinded comparison of wireless capsule endoscopy and multiphase CT enterography in obscure gastrointestinal bleeding. | Radiology, 2011;260(3): 744-751 | Comparing to CT |
| 56 | Wi J-H | Bowel preparation for capsule endoscopy: A prospective randomized multicenter study. | Gut and liver, 2009;3(3): 180-5 | The main outcome does not match. (Main outcome: Evaluation of Preparation) |
| 57 | Lapalus MG | Capsule endoscopy and bowel preparation with oral sodium phosphate: a prospective randomized controlled trial | Gastrointestinal endoscopy, 2008;67(7): 1091-1096 | The main outcome does not match. (Main outcome: Evaluation of Preparation) |
| 58 | Endo H | Ingesting 500 ml of polyethylene glycol solution during capsule endoscopy improves the image quality and completion rate to the cecum. | Digestive diseases and sciences, 2008; 53(12): 3201-5 | The main outcome does not match. (Main outcome: Evaluation of Preparation) |
| 59 | Guilhon de Araujo Sant'Anna AM | Wireless capsule endoscopy for obscure small-bowel disorders: final results of the first pediatric controlled trial. | Clinical gastroenterology and hepatology, 2005;3(3): 264-70 | Patients included in the study are limited. (Pediatric patients) |
| 60 | Choi EH | A multicenter, prospective, randomized comparison of a novel signal transmission capsule endoscope to an existing capsule endoscope | Gastrointestinal endoscopy, 2013;78(2): 325-32 | Comparing between two kinds of capsule endoscopes (Given PillCam SB and Medivators MiroCam) |
| 62 | Cave DR | A multicenter randomized comparison of the Endocapsule and the Pillcam SB. | Gastrointestinal endoscopy, 2008;68(3): 487-94 | Comparing between two kinds of capsule endoscopes (Given PillCam SB and Medivators MiroCam) |
| 63 | Jimenez-Garcia VA | Comparative study of three protocols to cathartic preparation for the study of colon by pillcam colon. | Gastroenterology, 2014;146(SUPPL. 1): S-771 | The main outcome does not match. (Main outcome: Evaluation of Preparation) |
| 64 | Choi E | A prospective double blinded randomized multicenter study comparing mirocam to pillcam SB2 in identifying sources of small intestinal bleeding | Am J Gastroenterol. 2011;106:S87 | Comparing between two kinds of capsule endoscopes (Given PillCam SB and Medivators MiroCam) |
| 65 | Robles, EPC | Emergency double-balloon enteroscopy combined with real-time viewing of  capsule endoscopy: A feasible combined approach in acute overt-obscure  gastrointestinal bleeding? | Digestive Endoscopy, 2015; 27(3):338-344 | Patients included in the study are limited. (Emergency patients) |
| 66 | Han, JW | Clinical Efficacy of Various Diagnostic Tests for Small Bowel Tumors and Clinical Features of Tumors Missed by Capsule Endoscopy | Gastroenterology research and practice, 2015:AR 623208 | Comparing to CT |
| 67 | Goncalves, TC | Small bowel capsule endoscopy in obscure gastrointestinal bleeding:normalcy is not reassuring | European journal of gastroenterology & hepatology, 2014;26(8):927-932 | The main outcome does not match. (Main outcome: Evaluation of Follow-up) |
| 68 | Min, CT | The role of double-balloon enteroscopy following capsule endoscopy in diagnosis of obscure Small intestinal diseases | Pakistan journal of medical science, 2013;29(2):479-484 | Comparing between Capsule endoscopy and Capsule endoscopy + Scope procedure. |
| 69 | Kawamura, H | Characteristics of the Small Bowel Lesions Detected by Capsule Endoscopy in Patients with Chronic Kidney Disease | Gastroenterology research and practice, 2013:814214 | Patients included in the study are limited. (Patients with Chronic Kindney Disease) |
| 70 | Harris, LA | Capsule Endoscopy in Patients with Implantable Electromedical Devices is Safe | Gastroenterology research and practice,2013:959234 | The main outcome does not match. (Main outcome: Safety of patients with Electro medical device) |
| 71 | Karargyris, A | Detection of Small Bowel Polyps and Ulcers in Wireless Capsule Endoscopy Videos | IEEE Trans Biomd eng, 2011;58(10):2777-86 | The main outcome does not match. (Main outcome: Data mining of capsule endoscope) |
| 72 | Szaflarska-Poplawska, A | Utility of capsule endoscopy in children and youth - own experience | Przreglad Gastroenerology 2012; 7(4):209-15 | Patients included in the study are limited. (Pediatric patients) |
| 73 | Bandorski, D | Capsule endoscopy in patients with cardiac pacemakers: Current situation in Germany | Zeitschr Gastroentercel gastroenterology, 2005;43(8):715-8 | Patients included in the study are limited. (Patient with pacemaker) |
| 74 | Chandran, S | Risk Stratification of Upper GI Bleeding With PillCam ESO Capsule | Gastrointestinal endoscopy, 2012;75(4): 256 | The main outcome does not match. (Main outcome: Evaluation of Risk Stratification in GI bleeding) |
| 75 | Choi, E | A Prospective Double Blinded Randomized Multicenter Study Comparing MiroCam to PillCam SB2 in Identifying Sources of Small Intestinal Bleeding | Am J Gastroenterol. 2011;106(2):S87 | Comparing between two kinds of capsule endoscopes (Given PillCam SB and Medivators MiroCam) |
| 76 | Gutkin, E | PillCam ESO (R) Is More Accurate Than Clinical Scoring Systems in Risk Stratifying Emergency Room Patients With Acute Upper Gastrointestinal Bleeding | Gastrointestinal endoscopy, 2010;71(5):157-8 | Patients included in the study are limited. (Patient in emergency room) |
| 77 | Pioche, M | Randomized Prospective Comparison of the Diagnostic Yield of PillCam SB2 degrees and Mirocam degrees Videocapsules in Patients With Obscure Digestive Bleeding | Gastrointestinal endoscopy, 2010; 71(5):AB123 | Comparing between two kinds of capsule endoscopes (Given PillCam SB and Medivators MiroCam) |
| 78 | Shalomov, A | Rapid Evaluation of the Upper GI Tract Using Real-Time Pillcam (TM) ESO in Emergency Room Patients with Acute UGI Bleeding | Am J Gastroenterol. 2009;104(3):S502 | Patients included in the study are limited. (Patient in emergency room) |
| 79 | Cave, DR | A multicenter randomized comparison of the Endocapsule and the Pillcam SB | Gastrointestinal endoscopy, 2008;68(3):487-94 | Comparing between two kinds of capsule endoscopes (Given PillCam SB and Olympus EndoCapsule) |
| 80 | Choi EH | A multicenter, prospective, randomized comparison of a novel signal,transmission capsule endoscope to an existing capsule endoscope. | Gastrointest Endoscopy. 2013 Aug;78(2):325-32 | Comparing between two kinds of capsule endoscopes (Given PillCam SB and Medivators MiroCam) |
| 81 | Purdy M | Characteristics of patients with a retained wireless capsule endoscope(WCE) necessitating laparotomy for removal of the capsule. | In Vivo. 2011 Jul-Aug;25(4):707-10 | The main outcome does not match. (Main outcome: Evaluation of Retention in GI bleeding) |
| 82 | Bechtel JF | Localizing an occult gastrointestinal bleeding by wireless PillCam SB capsule videoendoscopy in a patient with the HeartMate II left ventricular assist device. | J Thorac Cardiovasc Surg. 2010 Apr;139(4):e73-74 | Patients included in the study are limited. (Artificial heart) |
| 83 | Gan H | Sedation With Propofol Has No Effect on Capsule Endoscopy Completion, Rates: A Prospective Single-Center Study. | Medicine (Baltimore). 2015 Jul;94(27):e1140 | The main outcome does not match. (Main outcome: Evaluation of Sedation influence in GI bleeding) |
| 84 | Imagawa H | A trial of the use of patency capsules in combination with overnight capsule endoscopy. | Digestion. 2015;91(1):46-49. | The main outcome does not match. (Main outcome: Evaluation with Patency Capsule of in GI bleeding) |
| 85 | Hall BJ | A prospective 12-week mucosal healing assessment of small bowel  Crohn's disease as detected by capsule endoscopy. | Eur J Gastroenterol Hepatol. 2014 Nov;26(11):1253-9 | Patients included in the study are limited. (Crohn’s disease) |
| 86 | Oliva, Salvatore | Capsule endoscopy followed by single balloon enteroscopy in children with obscure gastrointestinal bleeding: A combined approach. | Digestive and Liver Disease, 2015;47(2):125-30 | Patients included in the study are limited. (Pediatric patients) |
| 87 | Fukushima, Masashi | A case series of Meckel's diverticulum: Usefulness of double balloon enteroscopy for diagnosis. | BMC Gastroenterology, 2014;14(1):arn. 155 | The main outcome does not match. (Main outcome: Evaluation of Meckel's diverticulum findings in GI bleeding) |
| 88 | Shiotani, Akiko | Application of fecal hemoglobin-haptoglobin complex testing for small bowel lesions | Scandinavian Journal of Gastroenterology, 2014;49(5): 539-44 | The main outcome does not match. (Main outcome: Evaluation of feacal Hb-Hpt complex testing findings in GI bleeding) |
| 89 | Lakatos, Peter Laszlo | Double balloon endoscopy for small intestinal disease: A single-center experience in Hungary | Medical Science Monitor, 2010;16(3):MT22-MT27 | The main outcome does not match. (Main outcome: Evaluation of Double balloon endoscopy in GI bleeding) |
| 90 | Gheorghe, Cristian | Olympus capsule endoscopy for small bowel examination. | J Gastrointestinal and Liver Diseases, 2007;16(3), 309-313 | The main outcome does not match. (Main outcome: Evaluation of Olympus EndoCapsule in GI bleeding) |
| 91 | Prasad, V. Mohan | Comparative study to determine the efficacy of the lateral viewing capsocam.RTM. capsule | American Journal of Gastroenterology, 2012;107(SUPPL. 1):S770 | The main outcome does not match. (Main outcome: Evaluation of Test device in GI bleeding) |

**2) Not Comparative**

|  | Author (Top) | Title | Journal | Reason of Exclusion |
| --- | --- | --- | --- | --- |
| 1 | Kurien M | Capsule endoscopy in adult celiac disease: a potential role in equivocal cases of celiac disease? | Gastrointest Endosc. 2013;Feb;77(2):227-32 | Single arm study of the capsule endoscopy |
| 2 | Pezzoli A | Interobserver agreement in describing video capsule endoscopy findings: a multicentre prospective study. | Dig Liver Dis. 2011;Feb;43(2):126-31 | Single arm study of the capsule endoscopy |
| 3 | Rubin M | Live view video capsule endoscopy enables risk stratification of patients with acute upper GI bleeding in the emergency room: a pilot study. | Dig Dis Sci. 2011 Mar;56(3):786-91 | Single arm study of the capsule endoscopy |
| 4 | Cheung DY | Capsule endoscopy in small bowel tumors: a multicenter Korean study. | J Gastroenterol Hepatol. 2010 Jun;25(6):1079-86 | Single arm study of the capsule endoscopy |
| 5 | Rondonotti E | Small bowel capsule endoscopy in clinical practice: a multicenter 7-year survey. | Eur J Gastroenterol Hepatol. 2010 Nov;22(11):1380-6 | Single arm study of the capsule endoscopy |
| 6 | Grève E | High diagnostic and clinical impact of small-bowel capsule endoscopy in patients with hereditary hemorrhagic telangiectasia with overt digestive bleeding and/or severe anemia. | Gastrointest Endosc. 2010 Apr;71(4):760-7 | Single arm study of the capsule endoscopy |
| 7 | Jang BI | Inter-observer agreement on the interpretation of capsule endoscopy findings based on capsule endoscopy structured terminology: a multicenter study by the Korean Gut Image Study Group. | Scand J Gastroenterol. 2010 Mar;45(3):370-4 | Single arm study of the capsule endoscopy |
| 8 | Katsinelos P | Wireless capsule endoscopy in detecting small-intestinal polyps in familial adenomatous polyposis. | World J Gastroenterol. 2009 Dec 28;15(48):6075-9. | Single arm study of the capsule endoscopy |
| 9 | Spada C | Polyethylene glycol plus simethicone in small-bowel preparation for capsule endoscopy. | Dig Liver Dis. 2010 May;42(5):365-70 | Single arm study of the capsule endoscopy |
| 10 | Van Gossum A | Capsule endoscopy versus colonoscopy for the detection of polyps and cancer. | N Engl J Med. 2009 Jul 16;361(3):264-70 | Single arm study of the capsule endoscopy |
| 11 | Postgate A | Feasibility of video capsule endoscopy in the management of children with Peutz-Jeghers syndrome: a blinded comparison with barium enterography for the detection of small bowel polyps. | J Pediatr Gastroenterol Nutr. 2009 Oct;49(4):417-23. | Single arm study of the capsule endoscopy |
| 12 | Postgate A | Are bowel purgatives and prokinetics useful for small-bowel capsule endoscopy? A prospective randomized controlled study. | Gastrointest Endosc. 2009 May;69(6):1120-8 | Single arm study of the capsule endoscopy |
| 13 | Papadopoulos AA | Effects of ageing on small bowel video-capsule endoscopy examination. | Am J Gastroenterol. 2008 Oct;103(10):2474-80 | Single arm study of the capsule endoscopy |
| 14 | Efthymiou A | Does clinical response correlate with mucosal healing in patients with Crohn's disease of the small bowel? A prospective, case-series study using wireless capsule endoscopy. | Inflamm Bowel Dis. 2008 Nov;14(11):1542-7 | Single arm study of the capsule endoscopy |
| 15 | Ross A | Double balloon enteroscopy detects small bowel mass lesions missed by capsule endoscopy. | Dig Dis Sci. 2008 Aug;53(8):2140-3 | Review article |
| 16 | Galmiche JP | Screening for esophagitis and Barrett's esophagus with wireless esophageal capsule endoscopy: a multicenter prospective trial in patients with reflux symptoms. | Am J Gastroenterol. 2008 Mar;103(3):538-45 | Single arm study of the capsule endoscopy |
| 17 | Iaquinto G | Capsule endoscopy is useful and safe for small-bowel surveillance in familial adenomatous polyposis | Gastrointest Endosc. 2008 Jan;67(1):61-7. | Single arm study of the capsule endoscopy |
| 18 | Christodoulou DK | Reproducibility of wireless capsule endoscopy in the investigation of chronic obscure gastrointestinal bleeding. | Can J Gastroenterol. 2007 Nov;21(11):707-14 | Single arm study of the capsule endoscopy |
| 19 | Sachdev MS | A prospective study of the utility of abdominal radiographs after capsule endoscopy for the diagnosis of capsule retention. | Gastrointest Endosc. 2007 Nov;66(5):894-900 | Prospective cohort study. |
| 20 | Wei W | Effect of mosapride on gastrointestinal transit time and diagnostic yield of capsule endoscopy. | J Gastroenterol Hepatol. 2007Oct;22(10):1605-8 | Single arm study of the capsule endoscopy |
| 21 | Estévez E | Diagnostic yield and clinical outcomes after capsule endoscopy in 100 consecutive patients with obscure gastrointestinal bleeding. | Eur J Gastroenterol Hepatol. 2006 Aug;18(8):881-8 | Single arm study of the capsule endoscopy |
| 22 | D'Halluin PN | Does the "Suspected Blood Indicator" improve the detection of bleeding lesions by capsule endoscopy? | Gastrointest Endosc. 2005 Feb;61(2):243-9 | Single arm study of the capsule endoscopy |
| 23 | Viazis N | Bowel preparation increases the diagnostic yield of capsule endoscopy: a prospective, randomized, controlled study. | Gastrointest Endosc. 2004 Oct;60(4):534-8 | Single arm study of the capsule endoscopy |
| 24 | Bailey AA | Diagnosis and outcome of small bowel tumors found by capsule endoscopy: a three-center Australian experience. | Am J Gastroenterol. 2006 Oct;101(10):2237-43. | Single arm study of the capsule endoscopy |
| 25 | Pennazio M | Outcome of patients with obscure gastrointestinal bleeding after capsule endoscopy: report of 100 consecutive cases. | Gastroenterology. 2004 Mar;126(3):643-53 | Single arm study of the capsule endoscopy |
| 26 | Ersoy O | How much helpful is the capsule endoscopy for the diagnosis of small bowel lesions? | World J Gastroenterol. 2006 Jun 28;12(24):3906-10. | Single arm study of the capsule endoscopy |
| 27 | Ang TL | Clinical utility, safety and tolerability of capsule endoscopy in urban Southeast Asian population. | World J Gastroenterol. 2003 Oct;9(10):2313-6. | Single arm study of the capsule endoscopy |
| 28 | Schlag C | Emergency video capsule endoscopy in patients with acute severe GI bleeding and negative upper endoscopy results. | Gastrointestinal endoscopy, 2015;81(4):889-95 | Single arm study of the capsule endoscopy |
| 29 | Eckardt AJ | Can inexperienced trainees reliably detect relevant clinical findings on capsule endoscopy and which mode and speed should they use? A randomized trial. | Gastrointestinal endoscopy, 2013; 77(5)SUPPL.1:AB269 | Single arm study of the capsule endoscopy |
| 30 | Sasmal, PK | Acute Small Intestinal Obstruction-An Unusual Complication of Capsule Endoscopy | Indian J surgery, 2015 ;77(SU 1), S21 | Single arm study of the capsule endoscopy |
| 31 | Watari, I | Is Occult Obscure Gastrointestinal Bleeding a Definite Indication for Capsule Endoscopy? A Retrospective Analysis of Diagnostic Yield in Patients with Occult versus Overt Bleeding | Gastroenterology research and paracice, 2013:915463 | Single arm study of the capsule endoscopy |
| 32 | Nutter, M | A Retrospective Analysis Comparing Small Bowel Follow-Through With Wireless | Gastroenterology nursing, 2010, 33(4):298- 302 | Single arm study of the capsule endoscopy |
| 33 | Shyung, LR | Capsule Endoscopy in Elderly Patients with Obscure Gastrointestinal Bleeding: Retrospective Analysis of 152 Cases | International journal of gerontology, 2010;4(1):23-27 | Single arm study of the capsule endoscopy |
| 34 | Kav, T | Clinical utility of capsule endoscopy in small intestinal diseases, experience of single referral center with 125 cases | Przeglad gastroenterologiczny, 2009;4(5):245-250 | Single arm study of the capsule endoscopy |
| 35 | Thameem, A | Intestinal polyposis in a 12 year old boy and role of Pillcam in diagnosis | Journal of gastroenterology and hepatology, 2011;26:251 | Single arm study of the capsule endoscopy |
| 36 | El Alaoui, M | Clinical impact of patients examined by PILLCAM SB (R) videocapsule: one-year follow-up of patients consecutively included in two French centers in 2003 | ACTA endoscopia, 2006;36(2):149-162 | Single arm study of the capsule endoscopy |

**3) Overlapped between premarketing and postmarketing**

|  | Author (Top) | Title | Journal | Reason of Exclusion |
| --- | --- | --- | --- | --- |
| 1 | Ohmiya N | Diagnosis and treatment of obscure GI bleeding at double balloon endoscopy | Gastrointestinal Endoscopy, 2007;66(3):S72-7. | Overlapped the study period between premarketing and postmarketing settings |

**4) Comparison with different patients between Capsule endoscopy and Flexible endoscopy**

|  | Author (Top) | Title | Journal | Reason of Exclusion |
| --- | --- | --- | --- | --- |
| 1 | Nakamura M | Preliminary comparison of capsule endoscopy and double-balloon enteroscopy in patients with suspected small-bowel bleeding. | Endoscopy, 2006;38(1):59-66. | Patients conducted Capsule endoscopy and Flexible endoscopy was different. |
| 2 | Fujimori S | Diagnosis and treatment of obscure gastrointestinal bleeding using combined capsule endoscopy and double balloon endoscopy: 1-year follow-up study. | Endoscopy. 2007;39(12):1053-8. | Patients conducted Capsule endoscopy and Flexible endoscopy was different. |
